# Inside Out: The Influence of Identity, Context and Time on Faculty Motivation and Engagement

**DOI:** 10.1111/tct.70280

**Published:** 2025-12-08

**Authors:** Kerri Shaffer, Sara M. Lamb, Candace J. Chow

**Affiliations:** ^1^ Graduate School of Education University at Buffalo Buffalo New York USA; ^2^ Spencer Fox Eccles School of Medicine University of Utah Salt Lake City Utah USA

## Abstract

**Introduction:**

Understanding why faculty engage in medical education is critical because it illuminates how to sustain faculty motivation and engagement. This study uses self‐determination theory (SDT) and practice theory to examine how faculty perceptions of their own competence, autonomy and relatedness evolve as external perceptions of skills, meaning and practice also change.

**Methods:**

We used longitudinal qualitative research to conduct a case study of nine faculty at one institution in the United States from AY2019–2020 through AY2023–24. Each faculty member was interviewed annually. Eight completed all interviews; one faculty left the study after the second year. After using reflexive thematic analysis to inductively code transcripts, we used theory to deductively code data.

**Results:**

As societal and institutional perceptions of competence, autonomy and relatedness changed over the 4 years of the study, faculty perspectives of these factors changed. As expertise in social determinants of health gained importance, faculty with this experience grew more confident about their teaching skills, while those with less lost confidence. All faculty experienced greater autonomy over drawing boundaries around personal lives with the advent of the COVID‐19 pandemic, but only the women faculty reflected on this during interviews. All experienced a loss of relatedness but found ways to connect virtually with each other and with students.

**Conclusion:**

Examining faculty engagement and motivation through SDT and practice theory with a longitudinal approach allowed us to understand how faculty perspectives about their roles as educators change over time and in relation to evolving contexts.

## 1 Introduction

An engaged and motivated faculty is key to ensuring the quality of higher education, as it relates to student success, scientific innovation and institutional visibility [1]. While there has been some study of faculty motivation in recent years, there remains a need for further exploration into how medical educators' individual differences and changing contexts influence their motivation and consequently their identities [1, 2]. Understanding the nuances of faculty motivation is important in supporting, training and retaining faculty from different backgrounds and with different skill sets [3, 4, 5].

In addition to the interplay of individual differences and context on faculty motivation, there is a growing body of evidence showing that faculty satisfaction and retention depend on developing an identity as an educator [6, 7, 8]. However, this identity is often elusive and difficult to develop for both structural and individual reasons [9]. The educational mission of an academic medical centre often receives less support than the clinical or research missions [9, 10, 11], which might limit professional identity development for educators. When structural support for education is lacking and other professional responsibilities compete with their educational roles, faculty identities as physician or scientist will remain more prominent [11]. Additionally, recognition, or lack thereof, is foundational to how faculty view themselves as educators and it is less common to receive recognition, funding, and leadership opportunities in the educational environment [6, 9, 11].

On a personal level, when multiple professional identities are not sufficiently integrated, physicians and scientists will struggle with the transition to education [6]. Because the educator role is seen as secondary to the primary role of physician or scientist, that identity is less well‐defined [11]. In turn, the educator identity may be perceived as a mere list of roles and responsibilities, rather than experienced on a more emotional level [11]. Finally, new medical educators are transitioning from fields where they are viewed as experts to an area where few have had formal training [12, 13]. This can lead to imposter syndrome, unrealistic expectations and a loss of self‐efficacy [9], which in turn can lead to a loss of motivation, as discussed in self‐determination theory (SDT).

Given the complex and ever‐changing nature of individuals, data on how best to support faculty motivation and their identity development as educators can be difficult to obtain through methods that limit examinations of phenomena to specific points in time and fail to consider the context in which those individuals practice. This study employed a longitudinal qualitative research (LQR) approach and used (SDT) in combination with practice theory to explore faculty motivation. Specifically, we asked the following: (1) How do evolving cultural perceptions of competence, autonomy and relatedness (internal factors) impact the ways that faculty identify as educators? And (2) how do evolving perceptions of skills, meaning and practice (external factors) affect their motivation and engagement?

## 2 Theoretical Framework

SDT has its roots in social psychology and is often employed to explore questions of motivation. It focuses on the intrinsic psychological needs of humans with relation to competence, autonomy and relatedness [14]. SDT posits that when extrinsic requirements are aligned with individual values and beliefs, they become internalised, prompting a willing and enthusiastic response, despite the motivation not originating intrinsically [15]. According to SDT, the conditions that must be in place to spark that internalisation are a sense of competency, autonomy and relatedness. If any of these are missing, motivation will remain extrinsic and engagement will wane [14].

While there is significant evidence of the efficacy of SDT in exploring issues of intrinsic motivation, its utility in studying the complex and dynamic nature of identity and the changing social environment is limited [16, 17]. While competence, autonomy and relatedness affect faculty motivation in specific contexts [18], the social identities of faculty and their access to privilege and power may influence how they experience them. To include these considerations and enrich the often universalist definitions that drive SDT, we used practice theory as an additional theoretical framework.

Practice theory began around the middle of the 20th century and has interdisciplinary roots in fields such as philosophy, anthropology, social theory, ethnomethodology and cultural theory [19]. Though there is no single agreed‐upon definition of practice, it is, in essence, an alternative to individualist approaches such as SDT [20]. In practice theory, the unit of analysis is not the individual but the field of practice, or array, of which the individual is a part, but not the centre [21]. Though the idea of what constitutes a practice varies widely, there are commonalities. All practice is embodied, in terms of both knowledge and ability, rooted in time and space, oriented toward an end, and held together by shared understanding [19]. These umbrella concepts can be simplified into three main components: materials, meaning and skills [22]. Taken together, these two theoretical lenses provide insight into both the intrinsic and extrinsic factors impacting faculty motivation.

## 3 Methods

### 3.1 Study Design

This study employed a longitudinal, qualitative case study design. LQR is used to collect qualitative data with the same participants through time to understand their journey [23, 24]. Case studies are used to study phenomena in particular, bounded contexts [25]. In this case, we examined experiences of medical educators at the SFESOM over the course of 4 years (AY2019–20 to AY2022–23). This study was deemed exempt by our university's Institutional Review Board. Participants provided consent prior to the first interview with the knowledge that quotes from interviews might be used in presentations/publications.

### 3.2 Context

The Spencer Fox Eccles School of Medicine (SFESOM), University of Utah, is a public institution with roughly 500 students. It is the only allopathic medical school in the state and is affiliated with the only academic health centre in the region. The SFESOM health system supports the delivery of healthcare for individuals in the surrounding states covering 10% of the geographic area of the United States. Our university is a predominantly white institution located in a historically White state, whose racial diversity is growing.

Our study also captured medical educators during a specific time in history. The second year of interviews was conducted a few months after the COVID‐19 pandemic had started and George Floyd's murder was captured on video and viewed by millions. Not only did our discussions provide opportunities to discuss education but we also discussed how educators were adjusting to virtual teaching and the United States' renewed interest in social and racial justice. In AY20–21, our institution launched a redesign of our medical education programme involving large‐scale curricular change, creation of new faculty, student, staff roles and an investment in new teaching methodologies.

### 3.3 Sampling and Participants

We invited faculty who had central administrative support and protected time to teach in our medical school programme (called core educators) to participate. In addition to receiving support, we provide professional development opportunities including retreats and writing sessions to motivate faculty in seeing themselves as educators. As maximum variation was a goal, we sought to include faculty with PhDs in foundational science and those with MD or DO degrees, faculty who were long‐time educators as well as those with less experience, those who identified as White as well as those who identified as people of colour, and faculty who were men and women (no one identified as non‐binary). Nine faculty joined the study, and eight faculty remained in the study for all 4 years. The ninth participant stopped participating after the second year. However, the composites below were written to capture the experiences of all nine participants. (Table 1)

**TABLE 1:** Participant demographics.

Rank	
Professor	2
Associate professor	5
Assistant professor	2
Gender	
Woman	4
Man	5
Degree	
MD/DO	5
PhD	4

### 3.4 Data Collection

CJC interviewed participants every summer preceding each academic year. The interviews lasted approximately 60 min. The first 2 years of interviews were conducted in person; the third and fourth were conducted via Zoom. All interviews were audio (or video) recorded and transcribed using Descript (Version 73.10, San Francisco, CA, USA). In the first year, we asked participants what being an educator meant, what they had learned about education and why they taught. In addition to returning to these questions, questions were added each year. In Year two, participants were asked about their social identities (e.g., race, socioeconomic status, gender and religion) and how social identities informed their professional work, if at all. In Year three, participants were asked to reflect on whether their understandings of their social identities had changed and what kind of self‐education they participated in to increase knowledge of their identities and positions in society. In the fourth year, participants were also asked about what success as an educator looked like to them.

### 3.5 Analysis Process

Data collection and the analysis process changed over time. Transcriptions were originally coded by CJC and a qualitatively trained research assistant using reflexive thematic analysis [26] with inductive codes including goals and motivations, intersection of social and professional identities, lessons learned and tensions. To analyse all 4 years of data together, we took a deductive approach. After we (CJC and KS) read the transcripts multiple times and saw that participants' sense of self and agency were central to their stories, we used SDT to examine how participant narratives aligned with concepts of autonomy, competency and relatedness. However, we soon found that those concepts could not fully account for the differences in the experiences, and consequently identities, of faculty who were otherwise similar in terms of education and training, so we added the subcodes *materials*, *meaning* and *skills* from practice theory to our primary SDT codes. Using both theories to guide analysis helped illuminate how each faculty's social and professional identity contributed to their different experiences of autonomy, competency and relatedness. We met each week during the coding process to share findings and insights.

We present the results by providing three composite narratives [27, 28, 29] followed by a comparison of the narratives to each other. A composite narrative ‘synthesizes the stories of research participants with the reflections and knowledge of the researcher to construct a storied account of the participants' combined experiences whilst maintaining their anonymity’ [29] (p. 22). Because we are telling the stories of participants through time, composite narratives also help us contextualise [29] the results in a way that presenting shorter thematic excerpts about specific timepoints cannot. In addition, composite narratives allow us to portray the nuances of identity development and the interconnections across participants' stories. Following the composites, we present comparisons of how the participants, as described in the composites, experienced motivation and engagement with respect to how their competence, autonomy and relatedness changed as the institution redefined what meanings, materials and skills over time.

*Composite narratives allow us to portray the nuances of identity development and the interconnections across participants' stories.*

### 3.6 Positionality and Reflexivity

Two of us have Director positions in our medical school's Dean's Office and one of us serves as Vice Dean of Education for the medical school. KS is Director of Curriculum and Faculty Support and identifies as a White woman. SML is a practicing physician and dean and identifies as a White woman. CJC is faculty and Director of Education Research and identifies as a woman of colour. We all know the participants well since we are all colleagues. We acknowledge that as qualitative researchers, we can only distance ourselves from our data so much and instead embrace the fact that the results presented below are coconstructions of our reflexivity and the participants' stories. A more detailed reflection on our experiences with LQR is documented in Gordon et al. [30]

## 4 Findings

**FIGURE 1:**
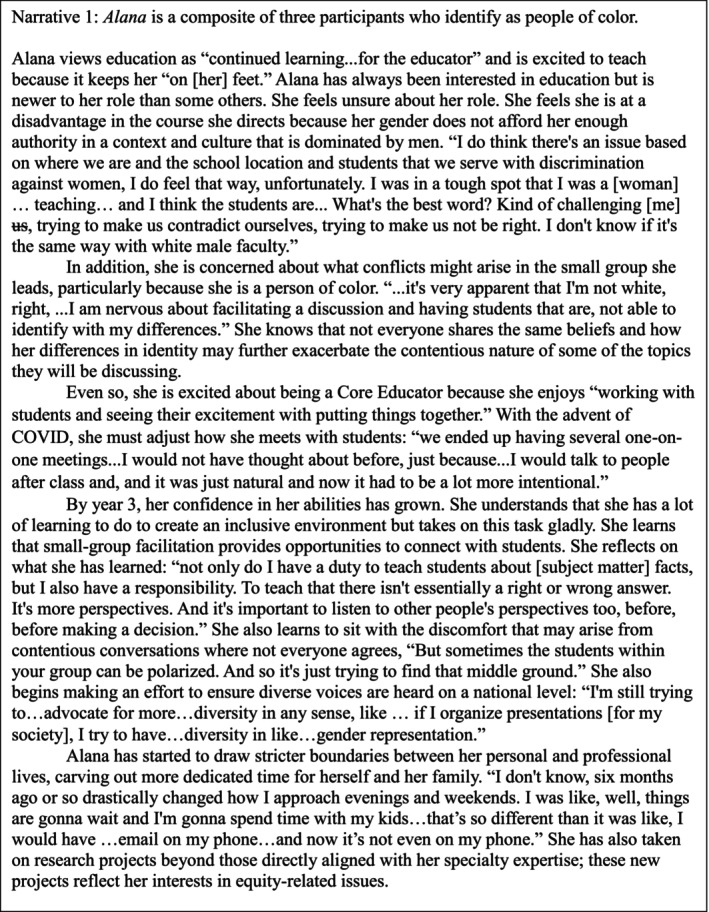
Alana's composite.

**FIGURE 2:**
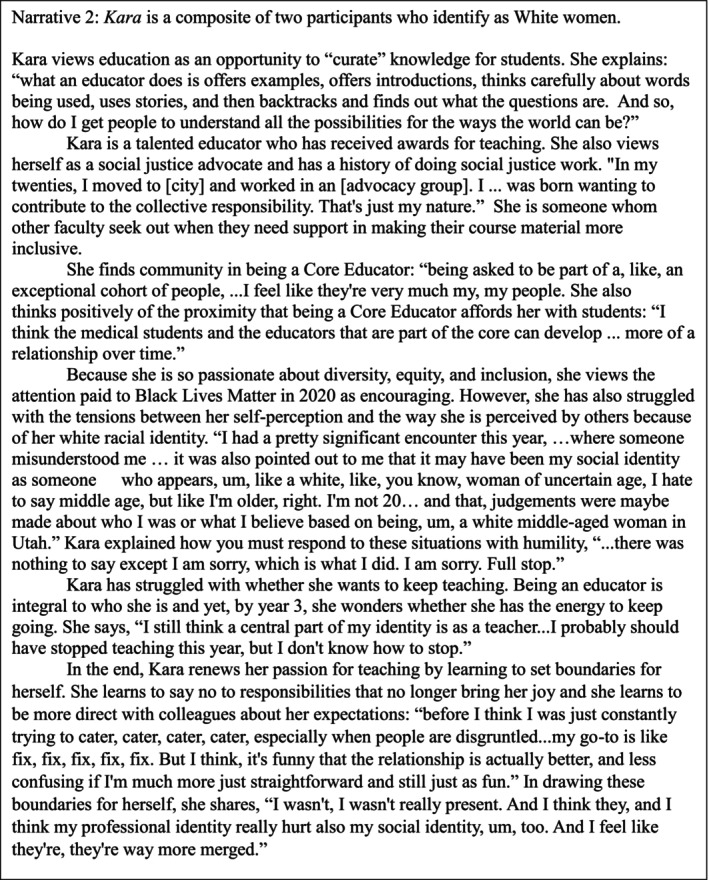
Kara's composite.

**FIGURE 3:**
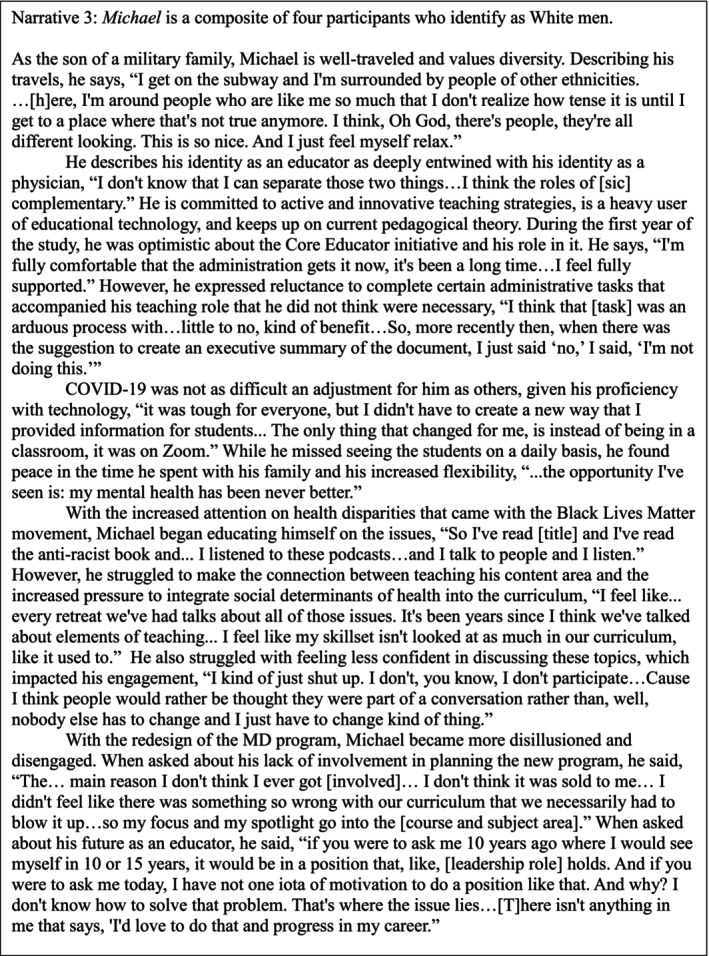
Michael's composite.

In Figures 1, 2 and 3, we present three composite narratives to describe how our participants think about education, their roles as educators and their social identities. Each is a combination of at least two participants. All quotes are original but quotes from multiple participants are used in combination to illustrate each composite's character. Following these figures is an analysis of how each participant's composite narrative illustrates how they have experienced motivation with respect to competency, autonomy and relatedness and how these experiences are related to their social identities and the evolving social context.

### 4.1 Analysis of Composites

Using the practice theory subcodes of *materials*, *meaning* and *skills* allowed us to see that the participants' experiences with competency, autonomy and relatedness did not evolve based on any explicit education or training, but rather on the changing cultural messages regarding what kind of expertise was most valuable in the current social and political context. We also found that their positionality with regard to race, ethnicity and gender impacted their professional identities and, consequently, their motivation and engagement with the programme's mission. Below, each section highlights how different aspects of practice (skills, meaning and materials) interacted with participants' identities via their perceptions of competence, autonomy and relatedness.

#### 4.1.1 Contextual Experiences With Competency

In all of these narratives, the motivation and engagement related to competency that each faculty member experienced was magnified or diminished depending on how well their skills aligned with cultural values. Michael's competence came largely from technical *skills*, which, in the beginning of the study and during the COVID‐19 pandemic, led to a strong sense of self‐efficacy. However, when diverse lived experiences and an understanding of advocacy and justice became more valued as areas of expertise, Alana and Kara were better positioned to be recognised for their *skills*. As a result, while Kara and Alana's motivation and engagement increased, Michael's decreased.

*In all of these narratives, the motivation and engagement related to competency that each faculty member experienced was magnified or diminished depending on how well their skills aligned with cultural values.*

When this study began, COVID‐19 was non‐existent, and George Floyd was still alive. Individuals like Alana were engaged in discussing topics like racism with students but were unsure of how such conversations would be received from a woman of colour. Alana's competence in leading such discussions grew as she gained experience and as general support for anti‐racist work and *skills* strengthened. This changing context allowed Alana's social identities and values to align more closely with her professional work, resulting in increased congruity between social and professional identities and motivation to teach.

Kara had a long history of teaching about social determinants of health (SDoH) and was bringing others on board by introducing them to *materials* she used and created. Her interest and s*kills* in SDoH were part of what motivated her to teach. However, although Kara found fulfillment in engaging with others around this area of competency, because others perceived Kara as a middle‐aged White woman from Utah, she was not automatically perceived as a social justice advocate. This discordance between her self‐perception and that of others caused tension for her professional identity and at times, prevented her from fully engaging in teaching.

Michael prided himself on being an excellent teacher, and recognition for these *skills* and *materials* he had created was motivating. Michael also viewed himself as a champion of diversity but did not see it as a major part of his teaching responsibilities. He sought out education on social justice issues including racism and understood that as a White man, he had much to learn about the experiences of people of colour. He also understood the need to diversify the student population to diversify the workforce. However, he saw this self‐education and need for increased diversity as separate from his responsibilities of teaching medical curricula. For Michael, these aspects of his social and professional identity were separate. While he previously felt very engaged with the core educator community, changing values forced him to wonder whether his *skills* and therefore his participation in the community were needed.

#### 4.1.2 Contextual Experiences With Autonomy

In the first year of the study, Alana and Kara struggled with setting boundaries and advocating for themselves, while Michael experienced support from his department and the education programme leadership. He enjoyed the freedom to be innovative and felt confident that the programme was moving in the right direction. As the study continued, these positions began to invert due to the changes in what was *meaningful* to the institution.

Alana and Kara reflected by the final year of interviews that they had much more confidence in saying no and drawing boundaries around their personal time. Because their *skills* were now more valuable to the institution and had increased *meaning*, they felt for the first time that they could step away from work when needed without any repercussions. Kara and Alana's ability to exert more autonomy is what motivated both of them to continue teaching. For Kara, letting go of some educational roles was energising, allowing her to re‐engage. For Alana, being able to choose research projects of interest allowed her to increase her engagement as an educator. Both Alana and Kara discussed this process of evolution and how much it had enhanced their well‐being. Conversely, Michael became more disillusioned as he felt his skills were less valued and he had less influence over the trajectory of the programme. He hesitated to participate in the creation of the new curriculum, the *meaning* of which was unclear to him. He found himself losing motivation and engagement, unsure of where his career was headed next.

*Because their* skills *were now more valuable to the institution and had increased* meaning*, they felt for the first time that they could step away from work when needed.*

#### 4.1.3 Contextual Experiences With Relatedness

Participant experiences with relatedness were similar. One of the goals of the Core Educator model was to build community among a core set of faculty. During the earlier interviews, all participants viewed the community as motivating and engaging. As Kara said, these were her ‘people’. However, they all experienced some loss during COVID, with respect to not seeing colleagues or students as regularly. Alana commented on the importance of engaging with others through Zoom meetings during this time, an example of how *materials* helped us to better contextualise the broader elements of SDT.

*Alana commented on the importance of engaging with others through Zoom meetings during this time, an example of how* materials *helped us better contextualise the broader elements of SDT.*

All participants appreciated the increased time with their families and the benefits it had on their mental well‐being. Kara, specifically, found power in the merging of her personal and professional lives and this improved her engagement and relationships at work. Alana worked within the constraints of social distancing to find ways of connecting with students, even as she increased the time she spent with her family. She went from being hesitant to discuss controversial topics with students to feeling more confident about teaching such topics, and this change in motivation affected her engagement with teaching and with students positively. Michael also reflected on how the flexibility introduced during the COVID‐19 pandemic afforded him more time with his family and improved his mental health. However, he felt his motivation and degree of engagement decrease with the advent of the new curriculum and focus on diversity, equity and inclusion because the *meaning* associated with his *skills* had changed.

## 5 Discussion

The participants had various understandings of themselves as educators and as individuals. These understandings changed through time, and as illustrated above, they changed with respect to a changing environment—first in response to a global pandemic, and afterwards with respect to a changing curriculum. Together, the narratives illustrate how context and identity influenced how participants experienced competence, autonomy and relatedness and as a result, motivation and engagement.

The field of medicine has a long history of privileging the expertise of and culture from White men. In recent years, educators and researchers have made progress in deconstructing this norm [31] by interrogating what and whose knowledge is important in health professions education [32]. The emphasis on understanding societal impacts on healthcare disparities was accelerated during the COVID‐19 pandemic, because of the inequitable impacts of COVID and because of the murders of several Black Americans [33]. While this resulted in bolstered competence and autonomy for our participants of colour, just as it empowered faculty of colour to speak out against the racism they had long witnessed and experienced [34, 35, 36], it seems to have had different effects for White individuals. In Kara's case, it highlighted tensions for White individuals involved in social justice work and in Michael's case, the question of whether he still had expertise to offer. This demonstrates the mutability of motivation in response to changes in the array of practice.

Community among a set of practitioners is important both for learning from and among peers [37] and for building relationships [38, 39]. The fact that this sense of community was lost during a period of social isolation is not unique to our institution. In response, like others in medical education [40, 41], our faculty learned they needed to be more intentional and flexible to sustain student engagement and communication [42]. They also created virtual opportunities to connect with and learn from each other, using the materials available to them.

Like others in the medical education community, our faculty experienced a shift of expectations around work and the ability to draw stricter boundaries around what they would and would not do [43]. The fact that the women participants spent more time discussing their efforts to create boundaries between personal and professional lives speaks to the impact of external gender dynamics on experiences of autonomy and relatedness [44].

As these results show, using practice theory in combination with SDT helped to enrich our exploration into the factors that influence faculty motivation. While the actual competence levels of participants in terms of universal teaching skills (e.g., facilitation and feedback, writing learning objectives and assessment and evaluation) likely increased in a measurable way over the 4 years of the study, their perceptions about what was meaningful played an equally important role in motivating them. The participants' responses to the social and programmatic changes demonstrated how faculty autonomy (both perceived and in reality) was influenced by social positions. This study helped to further illustrate the influence of the cultural environment on seemingly individual motivations and engagement [16, 18].

### 5.1 Implications for Practice

It could be assumed that the drivers of faculty motivation are universal and that recruitment, training and retention efforts can likewise be created with all in mind. However, as these composites show, motivation is multifaceted and faculty will have different experiences of competency, autonomy and relatedness informed by the interaction of individual skills, available resources and materials, social and professional identity and institutional perspectives on what is meaningful and valuable.

This awareness of the impact of identity and context on faculty motivation can help institutions be more intentional in their development of faculty, creating an environment that fosters psychological safety and active inclusion of marginalised voices. This can help ensure that the resources invested in faculty are used more effectively, with results that contribute to the continued quality of the programme and alignment with institutional values.

### 5.2 Limitations

This study was conducted at a specific institution and at a specific point in history. While these factors limit the scope of the findings, they also provide rich descriptions of faculty's journeys with their educator identities at a specific time and place. Our manuscript provides an overview of how the various contextual factors during this time (e.g., COVID‐19, remote learning, and a response to racially charged events) affected participants. If we had chosen to focus on just one of these aspects, like remote learning, we may have been able to highlight different elements of participants' competence, autonomy and relatedness with respect to skills, meaning and practice.

Another limitation is that the findings that we present are not necessarily the end of the participants' journeys. We expect their identities and levels of engagement to continue to evolve. In the United States, we are currently experiencing a backlash against various policies and programmes created in 2020 to enhance diversity, equity and inclusion, and if we had still been collecting data over the past year, it is likely this would have influenced participants' perceptions of their motivation and engagement as related to skills, materials and meaning.

## 6 Conclusions

It can be tempting to view these narratives as complete stories with a beginning, a middle and an end. However, this is precisely what LQR is *not* designed to do. The focus of LQR is on the journey *through time*, which is fluid and subjective, as opposed to *over time*, which implies a starting point and destination [23]. Rather, the value in LQR is its ability to show inter‐ and intra‐personal changes through time, juxtaposed against the social environment [45]. An approach to faculty engagement that takes into consideration the dynamic nature of context and identity may feel nebulous, but meeting faculty where and when they are will ultimately create the foundation for long‐term development and success. This study illustrates the interplay of time, context and identity, and their influence on faculty motivation, as well as the importance of recognising key cultural moments and leveraging them to create an environment that will support and sustain the identity formation of educators.

## Author Contributions

**Kerri Shaffer:** conceptualization, methodology, formal analysis, writing – original draft, writing – review and editing. **Sara M. Lamb:** conceptualization, methodology, writing – review and editing. **Candace J. Chow:** conceptualization, methodology, investigation, project administration, writing – original draft, writing – review and editing.

## Funding

The authors received no specific funding for this work.

## Conflicts of Interest

The authors declare no conflicts of interest.

## Data Availability

The data that support the findings of this study are available on request from the corresponding author. The data are not publicly available due to privacy or ethical restrictions.
